# Introductory of Prof. Brainard

**Published:** 1856-01

**Authors:** 


*Introductory of Prof. Brainard, on the occasion of the opening of the thirteenth Annual Session of the Rush Medical College.* Chicago, Ill.

This address contains the rise, progress, and history of that institution, from its origin down to the present time; the changes ot Faculty; the reasons for the reduction of fees and the history of other institutions which were projected around it.

We were not a little surprised, however, to find that the Professor has enumerated this department in his category of institutions which have "either proved entire failures, or been, at best, but partially successful." As all others mentioned have been "entire failures," we are left to console ourselves with the very gratifying announcement that we are "at best, but partially successful." We have the charity to believe that Prof. B. spoke from impressions *only*, and we hope certainly with no design or intention of giving this institution an indirect blow. We have too much confidence in the honorable feelings of that gentleman and his neighborly courtesy, to suppose that he would intentionally do us an injury, which he has been thus led inadvertently to do.

Six years since this institution commenced with an equal number of pupils which were found in the halls of the "Rush" in 1843–44. It is true that the number of pupils did diminish for three sessions, until, at the end of that time, there were but about fourteen. Up to this period, it suffered from a withering influence, from which at that juncture, it was happily relieved. The re-organization which followed this, was itself succeeded by continued success, the number of matriculants increasing with each session, until this session, the number of matriculants amount to nearly eighty, the precise number we cannot say, as at this moment the books of the Dean are not at hand. We may truthfully say that this department of the University never did enjoy health until three years ago, since which, it has been steadily growing and increasing.

Four years transpired before the "Rush Medical College" reached to seventy pupils, according to Dr. Brainard's own showing, and if he will allow us to date our rise from three sessions ago, we have traveled as rapidly as the institution with which he is connected. Indeed, the ratio of increase, calculating from our re-organization, would swell much higher than the numbers of their session of 1854–55. With this statement of facts, it will be seen that he does us injustice, (unintentionally, doubtless,) when he thus publicly declares that "at best, we are but partially successful," and therefore leave the matter for the present, after having called his attention to it, believing that he will at least do us the justice, in future, of correcting it.

